# PROTOCOL: Effects of lifestyle modification interventions to prevent and manage child and adolescent obesity: a systematic review

**DOI:** 10.1002/CL2.192

**Published:** 2018-11-02

**Authors:** Rehana A Salam, Jai K Das, Zahra Hoodbhoy, Karim Rizwan Nathani, Zulfiqar A Bhutta

## Background

### The problem, condition or issue

Obesity is a major public health crisis for children and adults across the world (Karnik 2012). Since 1980, the prevalence of overweight and obesity has increased remarkably in developed countries; 23.8% (22.9–24.7) of boys and 22.6% (21.7–23.6) of girls were overweight or obese in 2013, compared with 16.9% (16.1–17.7) of boys and 16.2% (15.5–17.1) of girls in 1980 (Ng 2014). Data from the National Health and Nutrition Examination Survey (NHANES) over a 25 year period (from 1988 to 2014) reported that in United States, obesity in children aged 2‐11 years continued to rise until 5‐8 years ago but has then plateaued (Ogden 2016). However, the trends in obesity in 12‐19 year olds continued to increase in a linear fashion (Ogden 2016). Similar trends were noted in the data obtained from the United Kingdom primary care electronic health record system, where obesity stabilized in the under 10 years olds but continued to rise in the adolescent population (van Jaarsveld 2015). Developing countries are now facing the “double burden of disease” (WHO 2017) which means that while undernutrition still remains a challenge, overweight and obesity is also rapidly increasing (WHO 2017). The prevalence of overweight and obesity in children and adolescents in low‐ and middle‐ income countries (LMIC), increased from 8.1% (7.7–8.6) in 1980, to 12.9% (12.3–13.5) in 2013 for boys and 8.4% (8.1–8.8) to 13.4% (13.0–13.9) in girls (Ng 2014). A comprehensive review of population based studies reported that obesity has accelerated in young girls and boys in Asian regions, Latin America and Africa (Abarca‐Gómez 2017). In contrast to the plateauing seen in the developed countries, the rise in mean body mass index (BMI) has been quite steep in east and south Asia (Abarca‐Gómez 2017). Obesity and overweight also lead to direct (medical) and indirect (nonmedical) costs that impose a significant economic burden to individuals and health care infrastructure. Twenty per cent of annual health care spending in the US is due to costs related to obesity (Spieker 2016) and this financial impact can have huge consequences in LMIC which have a fragile health system and mostly a lack of health insurance.(Agrawal 2016; Sartorius 2017).

There is considerable evidence to show that childhood obesity is a risk factor for type 2 diabetes, hypertension, coronary heart disease and stroke in adulthood (hazard ratios ranging from 1.1‐5.4) (Park 2012; Reilly 2011).

In 2004, the World Health Assembly adopted the “World Health Organisation (WHO) Global Strategy on Diet, Physical Activity and Health”, which encourages use of healthy diets and regular physical activity (WHO 2014). WHO developed the “Global Action Plan for the Prevention and Control of Non‐communicable Diseases 2013‐2020” which aims to achieve nine global non‐communicable diseases (NCD) targets by 2025, including a hold in rise of global obesity to match the rates of 2010. Obesity is caused by several modifiable and non‐modifiable risk factors (Ebbeling 2002). Non‐modifiable risk factors include biochemical and genetic factors (Koves 2017) while modifiable risk factors include sedentary behaviour, lack of physical activity and increased consumption of trans‐fat and sweetened products (Ebbeling 2002; Patrick 2004; Ebbeling 2002). Major recommendations from the World Health Assembly report on Ending Childhood Obesity (2016) include promoting intake of healthy foods; promoting physical activity; preconception and pregnancy care; early childhood diet and physical activity; health, nutrition and physical activity for school‐age children; and weight management (WHO 2016; WHO 2014). Numerous modalities have been developed to manage obesity including lifestyle modifications, pharmacological and surgical interventions (Al‐Khudairy 2017; Mead 2017; Oude 2009; Singh 2007; Waters 2011; WHO 2014). Pharmacological intervention consists of administration of drugs which assist weight loss while surgical methods include bariatric surgeries to assist weight loss mainly by reducing appetite. Lifestyle modifications include altering long‐term habits, typically of eating or physical activity, and maintaining the new behaviour. This review will focus on lifestyle interventions alone and will not include any pharmacological or surgical interventions for the prevention and management of obesity.

### The intervention

Various lifestyle, pharmacological and surgical interventions have been advocated to prevent and manage obesity in children and adolescents (Al‐Khudairy 2017; Mead 2017; Oude 2009; Singh 2007; Waters 2011; WHO 2014). For the purpose of this review, we will only review interventions related to lifestyle modifications. Lifestyle modification is defined as “altering long‐term habits, typically of eating or physical activity, and maintaining the new behaviour for months or years”. These interventions are largely implemented in school or community settings, where children and adolescents are available and follow up is convenient over a long period (Graf 2005; Hofsteenge 2014; Schranz 2014; Toulabi 2012; Vos 2011; Walpole 2013). Schools tend to be an ideal location for intervention due to the facilities available and easy access to this particular age group; they also offer time slots and equipment facility to encourage adequate physical activity (De Bourdeaudhuij 2011; Kobes 2018). Other delivery platforms include after‐school settings, sports club or within family settings (Kobes 2018). For the purpose of this review, we have categorised the lifestyle interventions as follows:

► Dietary intervention: Dietary interventions have been a mainstay for prevention and management of childhood and adolescent obesity. These interventions include dietary education and balanced meal provision.
  ▷ Dietary education: Educating children and/or their parents regarding healthy dietary behaviours has been a corner stone of several interventions that have targeted obesity in children and adolescents. It involves any combination of educational strategies, accompanied by environmental supports, designed to facilitate voluntary adoption of food choices and other food‐ and nutrition‐related behaviours conducive to health and well‐being. Nutrition lessons have been offered to participants along with their parents to motivate weight monitoring or reduction (Flodmark 1993; Hughes 2008; Johnston 2007b; Savoye 2007). The sessions are generally aimed at increasing awareness about the disadvantages of obesity in young age and the associated risks in adulthood as well as ways to achieve optimal weight. Interventions have also focused on encouraging skills for maintaining a food diary, weight charting, healthy cooking and organised shopping habits, which helps self‐monitoring and weight control (Braet 2004a; Epstein 1985; Epstein 1985a; Gillis 2007a; Kalavainen 2007; Nova 2001; Weyhreter 2003). Dietary education could be peer‐led, community‐based, parent‐oriented or through social media and communication platforms and could be a part of school curriculums. These are delivered by health workers, school teachers, trained peer, parents or community health workers.
  ▷ Provision of balanced meals (school or community based): Low dietary intake of fruits and vegetables is closely related to obesity and non‐communicable diseases (Glanz 2005). Multiple steps have been taken to promote increased consumption of fruits and vegetables and these include provision of balanced meals to children and adolescents in schools or in community settings. This intervention aims to reduce caloric input and provide balanced meals as a means to manage weight. Provision of balanced meals is one such intervention in which the nutrient composition can be altered to favour development during the growth phase in children and adolescents, while controlling total calorie intake to maintain body fat utilisation (Williams 2002). This is usually achieved by increasing percentage of proteins and decreasing carbohydrates and fats in diet (Ebbeling 2003; Rolland‐Cachera 2004; Williams 2002). Low glycaemic diets that may be achieved by reducing consumption of sugar‐sweetened beverages in children can reduce caloric intake and may lead to lower prevalence of obesity and related diseases (Al‐Khudairy 2017; Hu 2013; Kaiser 2013). Meals could be provided in schools or community set‐ups.
► Physical activity: This review will be addressing physical activity in two major categories: promoting exercise and reducing sedentary behaviour at home, school or community settings.
  ▷ Promoting physical exercise: Children and adolescents are encouraged to increase their physical movement to increase calorie expenditure through a certain combination of physical activities for a specific time. This might also include vigorous exercises in a gym or as sports, like swimming, soccer and basketball (Gutin 2002; Rolland‐Cachera 2004; Weintraub 2008). Aerobics and fitness drills have also been encouraged to increase calorie expenditure (Gutin 2002; Johnston 2007a). Simple outdoor activities, like walking are also be recommended (Rolland‐Cachera 2004).
  ▷ Reducing sedentary behaviour: Besides increasing physical activity, decrease in sedentary lifestyle has been related with reduced risk for metabolic syndrome and cardiovascular disease (Tremblay 2011). One such behaviour, television watching for more than 2 hours, has been shown to be associated with higher blood pressure and cholesterol level (Dasgupta 2006; Hancox 2004). Interventions that target restricting television and computer time in children have the potential to impact these unhealthy behaviours and prevent excessive weight gain (Viitasalo 2016).
► Behavioural therapy: Participants are encouraged to alter their behaviour and/or understanding towards their health parameters to promote healthy life. Studies have shown use of several therapies, like Cognitive Behavioural Therapy (CBT), Peer‐Enhanced Adventure Therapy (PEAT), Social Cognitive Therapy and Cue Exposure Therapy, to improve psychological awareness of current health and associated risks with obesity (Braet 2004; Duffy 1993; Jelalian 2006; Munsch 2008). The therapies can be offered only to participants, parents or both (Bogle 2011; Oude 2009; Waters 2011). Behavioural therapy is different from dietary education in the sense that behavioural therapy involves a comprehensive psychotherapeutic approach that aims to modify people's behavior and habits in line with their life goals and is usually delivered by a therapist.

### How the intervention might work

Lifestyle modifications can be effective in preventing and managing obesity along with its associated morbidity in children and adolescents (Al‐Khudairy 2017; Mead 2017; Oude 2009; Singh 2007; Waters 2011; WHO 2014). Lifestyle modifications can decrease calorie intake and/or increase the calorie expenditure of an individual (Camacho 2017). This tilts the balance towards utilisation of body's stored energy, in form of fats, causing weight loss. Loss of weight in overweight individuals results in reduced risk of developing chronic NCDs (Aucott 2005; Avenell 2004; Wing 2011). Individuals can have psychological benefit, in terms of self‐esteem and confidence, following loss of excessive weight and consequent relieve from social stigmatisation (Braet 2004; Roqué 2013).

**Dietary intervention:**

Dietary intervention intends to reduce total calorie consumption. This allows the body to utilise energy stored as fat, leading to weight loss. It plays a role in both the prevention as well as management of obesity. Long term adverse effects, like cholelithiasis, and gastrointestinal distress have been noticed with extremely low calorie diets which are hence is not recommended (Braet 2004). Diets corresponding to the food pyramid can be proposed for intervention (Braet 2004). Teaching healthy dietary habits and skills to children and parents, like healthy shopping or cooking may encourage a healthy diet (Braet 2004; Kalavainen 2007). Nutrition lessons increase the awareness of the participants and their parents about obesity and its associated risks (Johnston 2007). Improvement in knowledge may help motivate the participant to manage their weight.

Balanced diets can ensure an adequate supply of required nutrients during the growth phase in childhood and adolescence while maintaining negative energy balance to loose fats and assist weight management. Low glycaemic diet causes reduction of the overall consumption of energy rich items (Jelalian 2006). Discouraging fast food consumption will not only decrease the glycaemic load of the individuals but reduce the total calorie consumption (Rosenheck 2008). Lowering fast food consumption contributes to controlling obesity and its associated co‐morbidities (Jackson 2004; Rosenheck 2008).

**Physical activity:**

Theoritically, when the energy expenditure is equal to the energy intake, body weight should be maintained, which is the goal for preventing weight gain. However, if the intention is to loose weight, there is a need to decrease the energy intake and/or increase energy expenditure. Physical activity aims to increase calorie expenditure by the body. Increased calorie demand will lead to consumption of the stored excessive body fat and result in weight loss. It also avoids loss of lean muscle tissue in children and adolescent (Braet 2004). Exercise helps reduce the risk factors for cardiovascular disease by improving blood pressure, lipid profile and blood HbA1c levels (Gutin 2002). It results in decreased sedentary activities in participants, hence they are more active in their daily routine which leads to weight loss (Al‐Khudairy 2017). Sports activities, like swimming and basketball, as an entertainment can be very useful in encouraging participants for weight management (Rolland‐Cachera 2004; Weintraub 2008). Besides improving physical activities, reduction in sedentary behaviour, like television viewing, might also help engaging in more energetic activities that could lead to weight control (Bar‐On 2001; Hancox 2004).

**Behavioural therapy:**

Behavioural interventions develop the understanding and response of an individual towards obesity and health (Duffy 1993; Munsch 2008; Oude 2009). It encourages participants to self‐regulate and be compliant to their dietary habits and physical work to improve their weight management. Each behavioural therapy has its own method for psychological development of the participant. The individual is allowed to analyse his/her lifestyle from a more intellectual rather than emotional viewpoint. After understanding the importance of weight management, the participant self‐regulates his/her routine to support exercise and healthy nutrition. (Brennan 2013; Jelalian 2006). Self‐monitoring, through food diaries or weight charts, may encourage compliance of the individual to health habits for weight management (Gillis 2007).

### Why it is important to do the review

Obesity is associated with significant morbidity, hence accounting for a notable health care and economic burden (Ludwig 2009; Singh 2007). Multiple reviews and meta‐analyses exist evaluating the efficacy of interventions to prevent and manage obesity in children and adolescent. Table 1 summarises some of the relevant existing reviews related to the prevention and management of childhood and adolescent obesity. With the variety of interventions and the ever increasing number of reviews, it is difficult to generate conclusions regarding which interventions are relatively more effective compared to others for preventing and managing obesity in this age group. Moreover, the results of the existing meta‐analyses do not always point in the same direction, A recent meta‐synthesis has attempted moderator analyses to explain why some interventions are more effective than others; however it is limited to a few factors (Kobes 2018). Furthermore, the majority of the existing systematic reviews have restricted their inclusions to randomised controlled trials (RCTs) alone and focused too much on effectiveness aspect without focusing on the various contextual factors that might potentially impact the effectiveness of these interventions. Although RCTs are considered to be the gold standard when evaluating effectiveness, complementing RCT data with observational studies is sometimes imperative when evaluating complex lifestyle and behavioural interventions (Faraoni 2016; Black 1996; Hannan 2008; Oakley 2006; Stephenson 1998; Mulder 2018). Behavioural interventions are complex and influenced by various individual and environmental factors that could potentially impact the uptake and effectiveness of these interventions.

**Table 1:** Existing Systematic Reviews on Child and Adolescent Obesity

**Review Article**	**Target Population**	**Intervention reviewed**	**Primary Outcomes**	**Secondary Outcomes**	**No. of studies**	**Meta‐Analysis MD (95% CI)**
Oude 2009	Children and Adolescents Age (mean) &lt; 18 years	Drug Surgical Lifestyle ○ Diet ○ Physical Activity ○ Behavioral	BMI – SDS BMI z score Overweight (%age) Height Weight DXA BIA	Body Fat Distribution Metabolic markers Behavioral change Participants' views of intervention Self‐esteem Health Status Quality of life Cost effectiveness	64 RCTs	BMI‐SDS ○ Age &lt; 12 yrs ▪ 6 m FU: ‐0.06 (‐0.12, ‐0.01) ▪ 12 m FU: ‐0.04 (‐0.12, 0.04) ○ Age ≥12 yrs ▪ 6 m FU: ‐0.14 (‐0.17, ‐0.12) ▪ 12 m FU: ‐0.14 (‐0.18, ‐0.10)
Al‐Khudairy 2017	Adolescents Age 12 – 17 years	Lifestyle ○ Diet ○ Physical Activity ○ Behavioral	Change in BMI Change in BMI z score Change in weight Adverse events	Health‐related quality of life Self‐esteem All‐cause mortality Morbidity Anthropometric measures other than BMI Behavior change Participants' views of intervention Socioeconomic effects Parenting skills and relationships	44 RCTs	BMI change: ‐1.18 (‐1.67, ‐0.69) BMI z score change: ‐0.13 (‐0.21, ‐0.05) Weight change: ‐3.67 (‐5.21, ‐2.13)
Mead 2017	Children Age 6 – 11 years	Lifestyle ○ Diet ○ Physical Activity ○ Behavioral	Change in BMI Change in BMI z score Change in weight Adverse events	Health‐related quality of life Self‐esteem All‐cause mortality Morbidity Anthropometric measures other than BMI Behavior change Participants' views of intervention Socioeconomic effects	70 RCTs	BMI ○ change: ‐0.53 (‐0.82, ‐0.24) ○ z score change: ‐0.06 (‐0.10, ‐0.02) Weight change: ‐1.45 (‐1.88, ‐1.02)
Waters 2011	Children and Adolescent Age &lt; 18 years Mainly children were from Age 6 – 12 years	Lifestyle ○ Diet ○ Physical Activity ○ Behavioral	Weight and height Percentage fat content BMI Ponderal index Skin‐fold thickness Prevalence of overweight and obesity	Activity levels Dietary intake Change in knowledge Environment change (like food provision service) Stakeholders views of the intervention and other evaluation findings Self‐esteem Health status quality of life harm of the intervention cost effectiveness	55 studies Meta‐analysis included 37 studies	BMI/BMI z score: ○ All yrs: ‐0.15 (‐0.21, ‐0.09) ○ 0 – 5 yrs: ‐0.26 (‐0.53, 0.00) ○ 6 – 12 yrs: ‐0.15 (‐0.23, ‐0.08) ○ 13 – 18 yrs: ‐0.09 (‐0.20, 0.03)
Colquitt 2016	Children Age &lt; 6 years	Lifestyle ○ Diet ○ Physical Activity ○ Behavioral	Change in BMI/ BMI z score Change in weight Adverse events	Health related quality of life All‐cause mortality Morbidity Anthropometric measures (other than BMI) Behaviour change Participants' views of intervention Socioeconomic effects	7 RCTs	Change in BMI z score ○ ET: ‐0.26 (‐0.37, ‐0.16) ○ 6‐8 m PT: ‐0.38 (‐0.58, ‐0.19) ○ 12 m PT: ‐0.25 (‐0.40, ‐0.10)
Luckner 2011	Children and adults Age &lt; 18 years old	Lifestyle ○ Diet ○ Physical Activity ○ Behavioral	BMI	Body fat	68 controlled studies	Among children: ○ BMI with television viewing ▪ −0.27 (−0.4, −0.13) ○ BMI with lifestyle & education ▪ −0.1 >(−0.17, −0.04)
Liao 2014	Children Age &lt; 18 years	Lifestyle ○ Diet ○ Physical Activity ○ Behavioral	BMI	None	25 RCTs	Mean difference in BMI change from sedentary behaviour intervention ▪ −0.154; (−0.354, 0.045)
Vasques 2014	Children and adolescents Age <18 years	School based and after school interventions	BMI	None	52 studies	Effect size for change in BMI: 0.068 (P value &lt; 0.001)
Stice 2006	Children and adolescents Age <18 years	Lifestyle ○ Diet ○ Physical Activity ○ Behavioral	BMI	None	64 trials	21% of the trials showed significant effects

**Figure 1:**
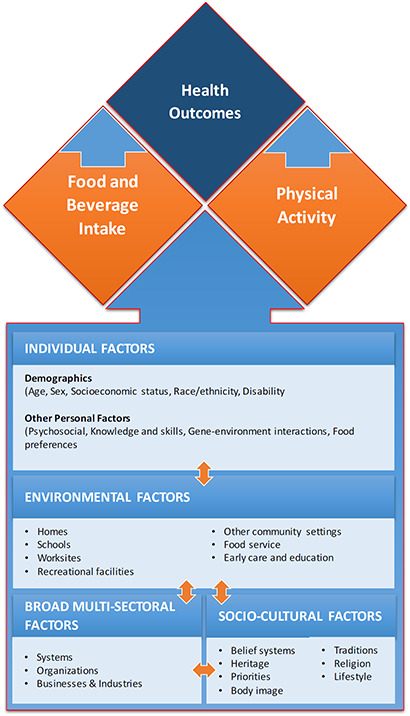
Theoretical frame work

We intend to assess the effectiveness of interventions along with various contextual factors that could potentially impact the uptake and effectiveness of these interventions based on the Social Ecological Model adapted from the Centers for Disease Control and Prevention (CDC) framework for addressing obesity disparities (CDC). Figure 1 depicts the logic model based on individual level factors (demographics, psychosocial, knowledge and skills, gene‐environment interactions and food preferences); environmental level factors (homes, early care and education, schools, worksites, recreational facilities, food service and retail establishments and other community settings); socio‐cultural level factors (belief systems, traditions, heritage, religion, priorities, lifestyle and body image); and broader multi‐sectoral level factors (government, organizations and business industries). In addition, we will also assess the studies included in our review with the lens of the PROGRESS framework (place, race, occupation, gender, religion, education, socioeconomic status, social status). This will provide important information that was in existing reviews. Firstly, it will provide a basis to guide public health program planners to adapt these interventions (alone or in combination) based on identifying factors that may affect how some groups engage with the intervention or the method of implementation. Secondly, this review will provide an insight to the public health researchers on whether outcomes differed by relevant socio‐demographic characteristics and whether the intervention was effective for disadvantaged groups. We will also assess whether studies included specific strategies to address diversity or disadvantage and whether the interventions help to decrease the gap between lower and higher income groups (O'Neill 2014). With the rapidly growing body of evidence in the field of obesity prevention and management and the recent development of the WHO action plan on NCDs (WHO 2013), it is important to comprehensively evaluate the evidence on effectiveness of all available interventions along with exploring the various contextual factors that might impact the effectiveness of these interventions. The proposed review will summarize up‐to‐date evidence for both children and adolescents with additional information regarding various contextual factors. This review can act as a single comprehensive body of evidence for policy makers and organizations that work in the area of childhood and adolescent obesity to plan programs to maximize the impact on the outcomes. It would allow us to suggest effective interventions for use in terms of age distribution, how and where the intervention was delivered, the resources (in terms of time and human resource) required to deliver the intervention and the cost‐effectiveness of each intervention (if available) and specific recommendations from the equity lens for LMIC, where the obesity burden is rising.

## Objectives

The objective of this review is to assess the impact of lifestyle interventions (including dietary interventions, physical activity, behavioural therapy or any combination of these interventions) along with the contextual factors to prevent and manage childhood and adolescent obesity.

## Methodology

### Criteria for including and excluding studies

#### Types of study designs

We will include primary studies using experimental and quasi‐experimental study designs that allow for causal inference. We will include non‐randomised studies with a control group in order to include large‐scale program evaluations so to capture important evidence on the effectiveness of lifestyle interventions from large scale programs and hence we would include the following study designs:

► Randomised controlled trials: Studies where participants are randomly assigned, individually or in clusters, to intervention and comparison groups
► Quasi‐experimental : Studies where non‐random assignment to intervention and comparison groups is based on other known allocation rules, including a threshold on a continuous variable (regression discontinuity designs) or exogenous geographical variation in the treatment allocation (natural experiments)
► Controlled before‐after (CBA) studies: Studies in which allocation to intervention and control groups was not made by study investigators, and outcomes were measured in both intervention and control groups at baseline and endline.
► Interrupted time series (ITS): Studies in which outcomes were measured in the intervention group at least three time points before the intervention was implemented and at least three time points after.

#### Types of participants

Participants will be children from 0‐9 years of age and adolescents 10‐19 years of age. We will include studies from both high income countries (HIC) and LMIC. Studies including only a subset of eligible participants will be included if the review provides separate results for the eligible subgroup. We will exclude studies conducted among hospitalised children and children and adolescents with any pre‐existing health conditions (e.g. diabetes, kidney disease, ADHD, Downs Syndrome etc.) because these types of interventions are not generalizable but provide information for the specific target groups.

#### Types of interventions

We will include the following interventions targeting children and adolescents:

► Dietary interventions
  ▷ Dietary education (school based, peer‐based, community based, social media, parent education)
  ▷ Provision of balanced meals (school or community)
► Physical activity
  ▷ Promoting physical exercise
  ▷ Reducing sedentary behaviour
► Behavioural Therapy
► Combination of any of the above interventions

We will analyse the above mentioned single interventions separately and studies assessing combinations of these interventions would be analysed separately. The above interventions could be compared against no intervention, standard of care (whatever is applicable in the study setting) or a combination of the above mentioned interventions. We will not include studies comparing one intervention with the other from the above list of intervention. In case of studies with multiple intervention arms, we will only include study arms that meet the inclusion criteria.

#### Types of outcome measures

*We will not use the list of outcomes as criteria for including studies.*

**Primary outcomes**

► BMI (defined as weight in kilograms (kg) divided by height in meters squared)
► BMI z‐scores (defined as a BMI measures of relative weight adjusted for child age and sex)
► Change in body weight (change in weight in kg)
► Adverse events (including symptoms associated with low calorie diet)

**Secondary outcomes**

► Prevalence of overweight and obesity
► Percentage body fat change
► Skin Fold Thickness
► Circumference
  ▷ Waist
  ▷ Hip
► Health‐related quality of life and self esteem (as defined by study authors)
► Cost effectiveness of the intervention

**Explanatory Secondary Outcomes:**

► Physical Activity intensity
► Total Caloric consumption

#### Duration of follow‐up

We will only include studies with a minimum duration of 12 weeks for intervention and follow‐up.

#### Types of settings

Studies conducted in any settings including schools, community or within family settings.

### Search strategy

We will use a comprehensive search strategy to identify eligible studies regardless of date of publication, language or publication status.

**Electronic searches**

We will search the following electronic databases: Cochrane Controlled Trials Register (CENTRAL), MEDLINE, EMBASE, CINAHL, PsycINFO, the WHO nutrition databases (http://www.who.int/nutrition/databases/en/), Social Science Index, and Dissertation Abstracts International. The trials registry Clinicaltrials.gov will be searched for ongoing trials. We will search Google along with key nutrition agencies databases such as Nutrition International, the Global Alliance for Improved Nutrition, the World Food Programme, and HarvestPlus to search for non‐indexed, grey literature to locate relevant programme evaluations and any additional trials.

**Searching other resources**

We will screen the reference lists of all included studies and relevant reviews to identify any additional trials that are not found by the electronic searches. We will attempt to obtain translations where possible if the review team is unable to translate particular papers.

### Description of methods used in primary research

For this review, the primary research design of interest will be experimental and quasi‐experimental study designs as well as non‐randomised studies with a control group, including CBA. We will also accept ITS studies with at least three time points before and three time points after the intervention. The studies will have participants aged 0 to 19 years exposed to pre‐specified lifestyle modification interventions including dietary interventions, physical activity, behavioural therapy or any combination of these interventions compared to no intervention or standard of care. One of the representative study of interest for this review is Singhal 2010. This study is a community based RCT assessing the effectiveness of a multi‐component intervention model of nutrition and lifestyle education on behavior modification, anthropometry and metabolic risk profile of urban Asian‐Indian adolescents in North India. The study included eleventh‐grade students aged 15–17 years from two co‐educational schools and were randomly assigned to intervention or control group. The intervention school received a multi‐component intervention including seven components of nutrition and lifestyle education and the adolescents in both the groups were assessed for behavior modification, anthropometry and metabolic risk profile.

### Criteria for determination of independent findings

Before initiating the synthesis (detailed below), we will ensure that all articles reporting on the same study are appropriately linked. To ensure independence and appropriate combination of outcome constructs, syntheses will be conducted according to the type of interventions specified above. If multi‐arm studies are included, intervention groups will be combined or separated into different forest plots, and we will ensure that there is no double counting of participants. If an outcome is reported in several different metrics, we will perform unit conversions in order to pool the data. We do anticipate differences in the types of literature and we will ensure that any analysis will take possible sources of dependency into account by grouping papers into studies and ensuring that no double counting of evidence takes place when synthesizing across studies.

### Selection of studies

Two review authors will independently assess potential study eligibility using predefined screening criteria. Any studies considered obviously irrelevant from screening the titles and/or abstracts will be excluded at the first level. Any uncertainties at the first level screening will be re‐assessed on the basis of full text in the second level of screening. For any discrepancies that may occur between the two reviewers, a third reviewer's opinion will be sought. We will resolve any disagreements through discussion until a consensus is reached. Reasons for exclusion of studies will be documented.

### Data extraction and management

Two review authors (ZH and KRN) will extract data independently and a third review author (JKD) will check for reliability and resolve any conflicts. We will check primary study data for accuracy and extract the following information in duplicate for each of the included studies:

► Background: time period when study took place, type of publication (e.g. full‐text journal article, abstract, conference paper, thesis), study country or countries, funding source(s), and conflicts of interest
► Population and setting: population age and setting
► Methods: Study design, description of study arms, unit of allocation, sample or cluster size per study arm (for individually or cluster randomised trials respectively), start and end date, follow up
► Participants: total number randomised, sample representativeness, baseline characteristics, number of withdrawals, socio‐demographic data.
► Intervention group details: number randomised to group, description of intervention, duration and follow‐up, timing, delivery of intervention, providers and their training. In case of studies with multiple intervention arms, we will describe all arms in the tables of included studies, while we will only report the arms that meet the inclusion criteria.
► Comparison group details: number randomised to group, description of comparison, duration and follow‐up, timing, providers and their training
► Outcomes: measurement tool, validation of the tool, total number in intervention and comparison groups, change indicated at each time point. In case if multiple measures are reported for the same outcome construct, we will use the one pre‐specified in our protocol.
► Contextual factors:
► Individual level factors: demographics, psychosocial, knowledge and skills, gene‐environment interactions and food preferences
► Environmental level factors: homes, early care and education, schools, worksites, recreational facilities, food service and retail establishments and other community settings
► Socio‐cultural level factors: belief systems, traditions, heritage, religion, priorities, lifestyle and body image
► Multi‐sectoral level factors: government, organizations and business industries.

Additionally, we will use PROGRESS (place, race, occupation, gender, religion, education, socioeconomic status, social status) checklist to record whether outcome data were reported by socio‐demographic characteristics known to be important from an equity perspective and to assess whether studies included specific strategies to address diversity or disadvantage (O'Neill 2014).

We will convert data to useable form and also contact authors for missing information. We will also examine any relevant retraction statements and errata for information.

### Assessment of risk of bias in included studies

Two review authors will independently assess quality of studies and risk of bias for each study. For randomised studies, we will use the Cochrane Risk of Bias tool recommended by the Cochrane Handbook for Systematic Reviews of Interventions (Higgins 2011), which assesses selection bias, performance bias, detection bias, attrition bias and reporting bias. We will rate each component as ‘high’, ‘low’, or ‘unclear’ for each risk of bias component. For non‐randomised studies, we will use the Cochrane Effective Practice and Organisation of Care (EPOC) risk of bias criteria (based on additional criteria including similar baseline outcome measurements, similar baseline characteristics, knowledge of the allocated interventions adequately prevented during the study, protection against contamination, intervention independent of other changes, shape of intervention effect pre‐specified and intervention unlikely to affect data collection) and rate the studies as low risk, high risk or unclear risk (EPOC 2017). We will provide supporting evidence for the risk of bias judgements. Two independent reviewers will perform quality appraisal for each study and disagreements will be resolved by discussion or consultation with a third reviewer.

We will summarise the quality of evidence according to the outcomes as per Grading of Recommendations, Assessment, Development and Evaluation (GRADE) criteria (Walker 2010). A grade of ‘high’, ‘moderate’, ‘low’ and ‘very low’ will be used for grading the overall evidence indicating the strength of an effect on specific health outcome based on methodological flaws within the component studies, consistency of results across different studies, generalizability of research results to the wider patient base and how effective the treatments have been (Balshem 2011). For non‐randomised studies, the evidence quality will be updated based on large magnitude of effect, dose response and effect of all plausible confounding factors would be to reduce the effect (where an effect is observed) or suggest a spurious effect (when no effect is observed). Two reviewers will discuss ratings and reach consensus, and disagreements will be resolved by consulting a third reviewer. We will develop a summary of findings table to show the effects for the primary outcomes.

### Measures of treatment effect

We will perform statistical analysis using RevMan 5 (Revman 2014). We will analyse dichotomous data using risk ratio (RR) with 95% confidence intervals (CI). For continuous data, we will use the mean difference (MD) with 95% CI, if outcomes are measured in the same way between trials. We will use the standardized mean difference (SMD) with 95% CI to combine trials that measure the same outcome but use different methods of measurement.

### Unit of analysis issues

Where trials have used clustered randomisation, we anticipate that study investigators would have presented their results after appropriately controlling for clustering effects (for example, variance inflated standard errors, hierarchical linear models). If it is unclear whether a cluster‐ randomised controlled trial has appropriately accounted for clustering, the study investigators will be contacted for further information. Where appropriate controls for clustering were not used, we will request an estimate of the intra‐class correlation coefficient. The data will be re‐analysed using multi‐level models which control for clustering. Following this, effect sizes and standard errors will be meta‐analysed in RevMan using the generic inverse method (Higgins 2011a). They will be combined with estimates from individual level trials. We will use sensitivity analyses to assess the potential biasing effects of using the interclass correlation coefficients that have been derived in different ways.

### Dealing with missing data

If the outcome of interest does not include data on all participants, we will first contact the trial authors via email to inquire about data for the missing cases. Missing data if found will be re‐included in the analysis. If unable to find missing data we will analyse data for only those participants whose results are available, and address the impact of the missing data in the assessment of risk of bias.

### Assessment of heterogeneity

We will assess heterogeneity among studies in two ways. Firstly, we will assess heterogeneity at face value: heterogeneity in population, interventions, or outcomes. Secondly, heterogeneity between trial results will be tested using a standard Chi² test, to assess whether observed differences in results are compatible with chance alone (Higgins 2011). We will report statistical heterogeneity as I^2^, Q, and tau^2^ for all random‐effects meta‐analyses.

### Assessment of reporting biases

If there are 10 or more studies in a meta‐analysis, we will investigate reporting biases using funnel plots. If the plots are asymmetrical, we will consider various explanations such as publication bias, poor study design etc.

### Data synthesis

We will pool data from studies we judge to be clinically homogeneous. If more than one study provides usable data in any single comparison, we will perform a meta‐analysis. We will conduct separate meta‐analysis based on the interventions (dietary intervention, physical activity, behavioural therapy or any combination of these). Separate meta‐analysis will be conducted for each intervention, outcome and study design (RCT, ITS and CBA). We will standardize all the reported effect sizes as RRs for the dichotomous outcome and synthesize all effect sizes as a common metric and synthesized together.

We will carry out statistical analysis using the Review Manager software (Revman 2014). We will use random‐effects meta‐analysis for combining data to produce an overall summary, since we expect reasonable clinical heterogeneity in interventions, comparisons, outcomes, or settings within the studies included. The random‐effects summary will be treated as the average of the range of possible treatment effects and we will discuss the clinical implications of treatment effects differing between trials. If the average treatment effect is not clinically meaningful, we will not combine trials.

For each comparison, we will descriptively summarise the findings from the contextual factors at the individual, environmental, socio‐cultural and multi‐sectorial levels based on our logic model to assess their impact on the implementation and effectiveness of each intervention.

### Subgroup analysis and investigation of heterogeneity

Where data will allow, we will perform the subgroup analyses listed below, to explore substantial and considerable heterogeneity across studies:

► Age (e.g. 0‐9 year versus 10 to 19 years of age)
► HIC versus LMIC
► Duration or intensity of intervention (e.g. short versus long term, one‐off versus multiple sessions)
► Study setting, school, community etc.
► Methodological quality of the included studies (Low risk of bias versus high risk of bias)

We will assess difference in subgroups based on the methodology described in the Cochrane Handbook (Higgins 2011) by using a simple approach for a significance test to investigate differences between two or more subgroups. We will undertake a standard (Chi‐Square) test for heterogeneity across subgroup results rather than across individual study results. We will report the I^2^ statistic for subgroup differences that describes the percentage of the variability in effect estimates from different subgroups due to genuine subgroup differences rather than sampling error. We will not conduct meta‐regression.

### Sensitivity analysis

The potential effects of biases on the quality of the included trials will be assessed by sensitivity analysis. We will exclude studies at high risk for selection and attrition bias (> 20%) from the analysis to assess the difference on the pooled result.

### Treatment of qualitative research

We do not plan to include qualitative research.

## Annexure

### Search strategy (MEDLINE)

1 SU adolescent OR SU child OR SU infant OR kid* OR child* OR pediat* OR baby OR babies OR infan* OR paed* OR pediat* OR teen*
2 adolescen* OR pubescen* OR juvenil* OR youth OR young* OR pubert* OR preschool* OR toddler*
3 1 OR 2
4 SU obesity OR SU pediatric obesity OR SU overnutrition OR SU overweight OR SU body mass index OR SU body weight changes OR obes* OR overweigh* OR over weigh* OR overnutrition OR over nutrition OR increas* weigh*
5 weigh* gain* OR weigh* loss OR over‐weigh* OR over‐nutriti* OR fat* OR body fat* OR BMI OR Body mass index OR circumferenc*
6 4 OR 5
7 SU psychotherapy OR SU motor activity OR SU exercise OR SU diet OR SU eating OR SU food OR behavio* therap* OR counsel* OR famil* therap* OR soci* support* OR exercis* OR exercis* therap*
8 physical* educat* OR health promot* OR lifestyle OR healthy nutrit* OR educat* OR physical* OR activ* OR walk* OR jog* OR swim* OR danc* OR weigh* lift*
9 aerobic* OR train* OR aware* OR behav* educat* OR physical* activ* OR diet* OR diet* therap* OR health* educat* OR diet* educat* OR nutrition* OR food* OR eat*
10 food intake OR overeating OR balance* meal OR balance* diet* OR physical* train* OR game* OR psychotherap* OR peer* support* OR group therap* OR cognit* therap* OR psycholog* therap*
11 7 OR 8 OR 9 OR 10
12 3 AND 6 AND 11
13 randomi* control* trial OR Quasi randomi* OR Quasirandomi* OR Quasi‐randomi* OR before after Stud* OR before‐after Stud* OR Interrupt* time ser* OR control* Stud* OR control* trial OR randomi* OR placebo OR control* group
14 12 AND 13

## Review authors

**Lead review author:** The lead author is the person who develops and co‐ordinates the review team, discusses and assigns roles for individual members of the review team, liaises with the editorial base and takes responsibility for the on‐going updates of the review.

**Table:** 

Name:	Rehana A Salam
Title:	Ms.
Affiliation:	South Australian Health and Medical Research Institute, University of Adelaide and Aga Khan University
Address:	University of Adelaide, Australia
City, State, Province or County:	Adelaide
Post code:	5000
Country:	Australia
Phone:	451321440
Email:	rehana.salam@aku.edu
**Co‐authors:**	
Name:	Jai K Das
Title:	Dr
Affiliation:	Aga Khan University
Address:	Stadium Road
City, State, Province or County:	Karachi
Post code:	74800
Country:	Pakistan
Phone:	+92‐21‐34930051
Email:	jai.das@aku.edu
	
Name:	Karim Rizwan Nathani
Title:	Dr.
Affiliation.	Research Associate, Division of Women and: Child Health, Department of Paediatric, The Aga Khan University
Address:	Stadium Road, Karachi
City, State, Province or County:	Karachi, Sindh
Post code:	74800
Country:	Pakistan
Phone:	+92 333 2178293
Email:	karim.rizwan.n@gmail.com
	
Name:	Zahra Hoodbhoy
Title:	Dr.
Affiliation:	Division of Women and Child Health The Aga Khan University
Address:	Stadium Road PO Box 3500
City, State, Province or County:	Karachi
Post code:	74800
Country:	Pakistan
Phone:	92‐21‐34864126
Email:	zahra.hoodbhoy@aku.edu
	
Name:	Zulfiqar A Bhutta
Title:	Dr
Affiliation:	Centre for Global Child Health, The Hospital for Sick Children, Toronto, Canada and Centre of Excellence in Women and Child Health, Aga Khan University, Karachi, Pakistan.
Address:	SickKids
City, State, Province or County:	Toronto
Post code:	M5G 1X8
Country:	Canada
Phone:	416‐813‐7654 ext. 328532
Email:	zulfiqar.bhutta@sickkids.ca

## Roles and responsibilities

Please give a brief description of content and methodological expertise within the review team. It is recommended to have at least one person on the review team who has content expertise, at least one person who has methodological expertise and at least one person who has statistical expertise. It is also recommended to have one person with information retrieval expertise. Please note that this is the *recommended optimal* review team composition.

- Content: Rehana A Salam (RAS), Jai K Das (JKD) and Zulfiqar A Bhutta (ZAB)
- Systematic review methods: RAS, JKD
- Statistical analysis: RAS, JKD, Zahra Hoodbhoy (ZH), Karim Rizwan Nathani (KRN)
- Information retrieval: ZH, KRN

## Sources of support

Describe the source(s) of financial and other support for the proposed review.

## Declarations of interest

None to declare: RAS, JKD, ZH, KRN, ZAB

## Preliminary timeframe

Approximate date for submission of the systematic review. 31 January 2019

## Plans for updating the review

Reviews should include in the protocol specifications for how the review, once completed, will be updated. This should include, at a minimum, information on who will be responsible and the frequency with which updates can be expected.

Rehana A Salam will be responsible for updating this review and the review will be update every two years after publication date.

## AUTHOR DECLARATION

### Authors' responsibilities

By completing this form, you accept responsibility for preparing, maintaining and updating the review in accordance with Campbell Collaboration policy. Campbell will provide as much support as possible to assist with the preparation of the review.

A draft review must be submitted to the relevant Coordinating Group within two years of protocol publication. If drafts are not submitted before the agreed deadlines, or if we are unable to contact you for an extended period, the relevant Coordinating Group has the right to de‐register the title or transfer the title to alternative authors. The Coordinating Group also has the right to de‐register or transfer the title if it does not meet the standards of the Coordinating Group and/or Campbell.

You accept responsibility for maintaining the review in light of new evidence, comments and criticisms, and other developments, and updating the review at least once every five years, or, if requested, transferring responsibility for maintaining the review to others as agreed with the Coordinating Group.

### Publication in the Campbell Library

The support of the Coordinating Group in preparing your review is conditional upon your agreement to publish the protocol, finished review, and subsequent updates in the Campbell Library. Campbell places no restrictions on publication of the findings of a Campbell systematic review in a more abbreviated form as a journal article either before or after the publication of the monograph version in Campbell Systematic Reviews. Some journals, however, have restrictions that preclude publication of findings that have been, or will be, reported elsewhere and authors considering publication in such a journal should be aware of possible conflict with publication of the monograph version in Campbell Systematic Reviews. Publication in a journal after publication or in press status in Campbell Systematic Reviews should acknowledge the Campbell version and include a citation to it. Note that systematic reviews published in Campbell Systematic Reviews and co‐registered with Cochrane may have additional requirements or restrictions for co‐publication. Review authors accept responsibility for meeting any co‐publication requirements.

**I understand the commitment required to undertake a Campbell review, and agree to publish in the Campbell Library. Signed on behalf of the authors**:

**Table:** 

**Rehana A Salam**	**October 31, 2018**
**Form completed by:**	**Date:**
